# Antimicrobial Peptides: Primeval Molecules or Future Drugs?

**DOI:** 10.1371/journal.ppat.1001067

**Published:** 2010-10-28

**Authors:** Brian M. Peters, Mark E. Shirtliff, Mary Ann Jabra-Rizk

**Affiliations:** 1 Graduate Program in Life Sciences, Microbiology and Immunology Program, School of Medicine, University of Maryland, Baltimore, Maryland, United States of America; 2 Department of Microbial Pathogenesis, Dental School, University of Maryland, Baltimore, Maryland, United States of America; 3 Department of Microbiology and Immunology, School of Medicine, University of Maryland, Baltimore, Maryland, United States of America; 4 Department of Oncology and Diagnostic Sciences, University of Maryland–Baltimore, Maryland, United States of America; University of California San Francisco, United States of America

## Antimicrobial Peptides: Effector Substances of Innate Immunity

From the outside and within, we are constantly bombarded with a myriad of diverse microbial species. However, our bodies are equipped with an evolutionarily conserved innate immune defense system that allows us to thwart potential pathogens. Antimicrobial peptides (AMPs) are a unique and assorted group of molecules produced by living organisms of all types, considered to be part of the host innate immunity [1], [2]. These peptides demonstrate potent antimicrobial activity and are rapidly mobilized to neutralize a broad range of microbes, including viruses, bacteria, protozoa, and fungi [3]. More significantly, the ability of these natural molecules to kill multidrug-resistant microorganisms has gained them considerable attention and clinical interest [1]. With the growing microbial resistance to conventional antimicrobial agents, the need for unconventional therapeutic options has become urgent. This article provides an overview of AMPs, their biological functions, mechanism of action, and applicability as alternative therapeutic agents.

## Structure and Classification

**Figure 1:**
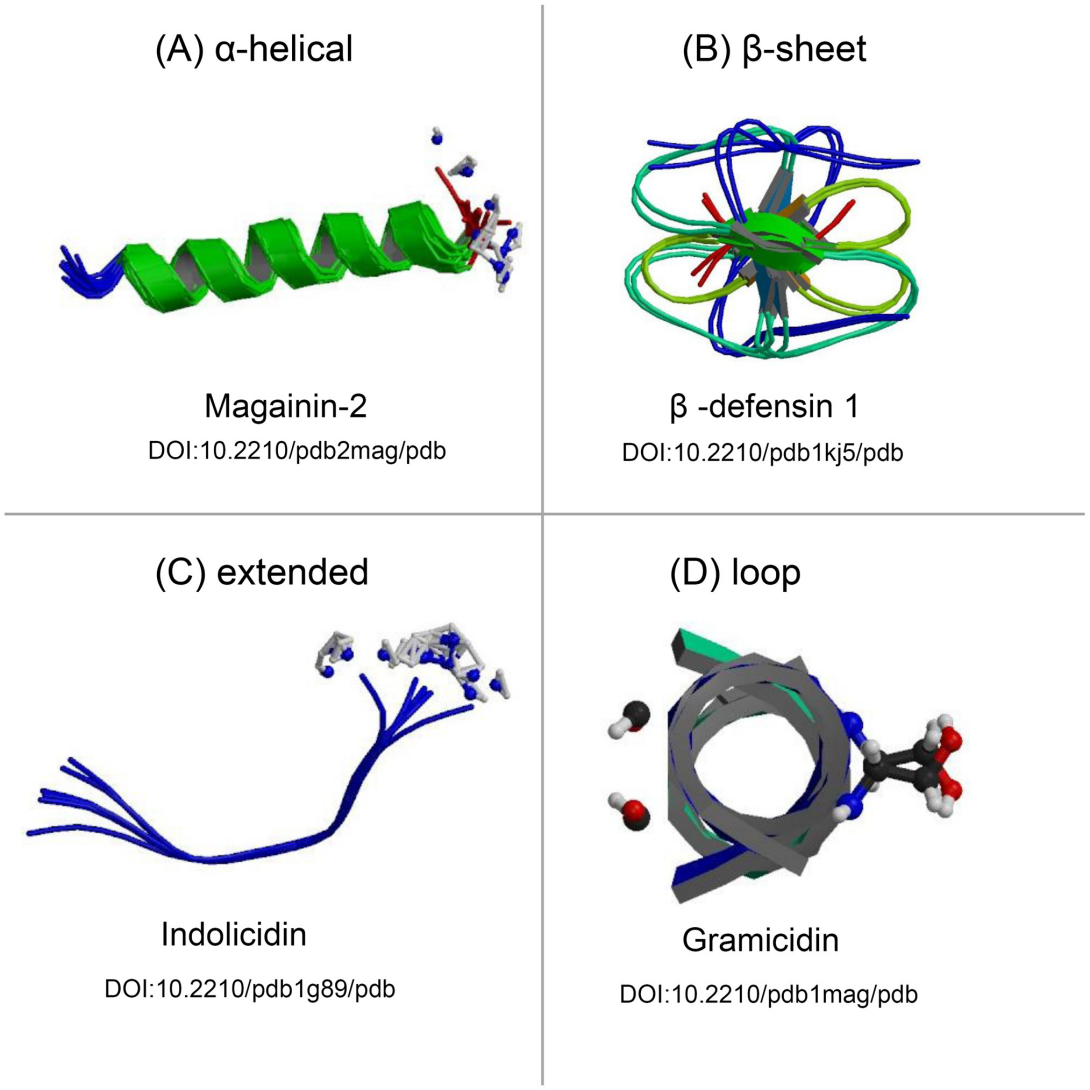
Protein models representing the structural differences of the four classes of antimicrobial peptides. Antimicrobial peptides can be grouped into four major classes based on their secondary structures, including the (A) α-helical peptides, (B) peptides composed of a series of β-sheets, (C) peptides that adopt unconventional structures, such as extended helices, and (D) peptides that assemble into loops. All structures were obtained freely from the RCSB Protein Data Bank (PDB) (http://www.pdb.org/) and have been referenced according to their Digital Object Identifier (DOI) [23]. Additional information for each AMP may be obtained by consulting the RCSB PDB and cross-referencing the DOI.

Antimicrobial peptides are small, positively charged, amphipathic molecules (which possess both hydrophobic and hydrophilic regions) of variable amino acid composition and length (six to 100 amino acids). Based on their secondary structure, AMPs are grouped into four major classes: β-sheet, α-helical, loop, and extended peptides (Figure 1) [1]. Currently, more than 800 natural AMPs with several different sequences have been isolated from a wide range of organisms (Antimicrobial Peptide Database, http://aps.unmc.edu/AP/main.php). In humans, the most prominent innate AMPs are the cathelicidins and defensins produced primarily by cells of the immune system and the histatins produced and secreted into the saliva by the parotid, mandibular, and submandibular salivary glands [4]–[6].

## Mechanism of Antimicrobial Activity

**Figure 2:**
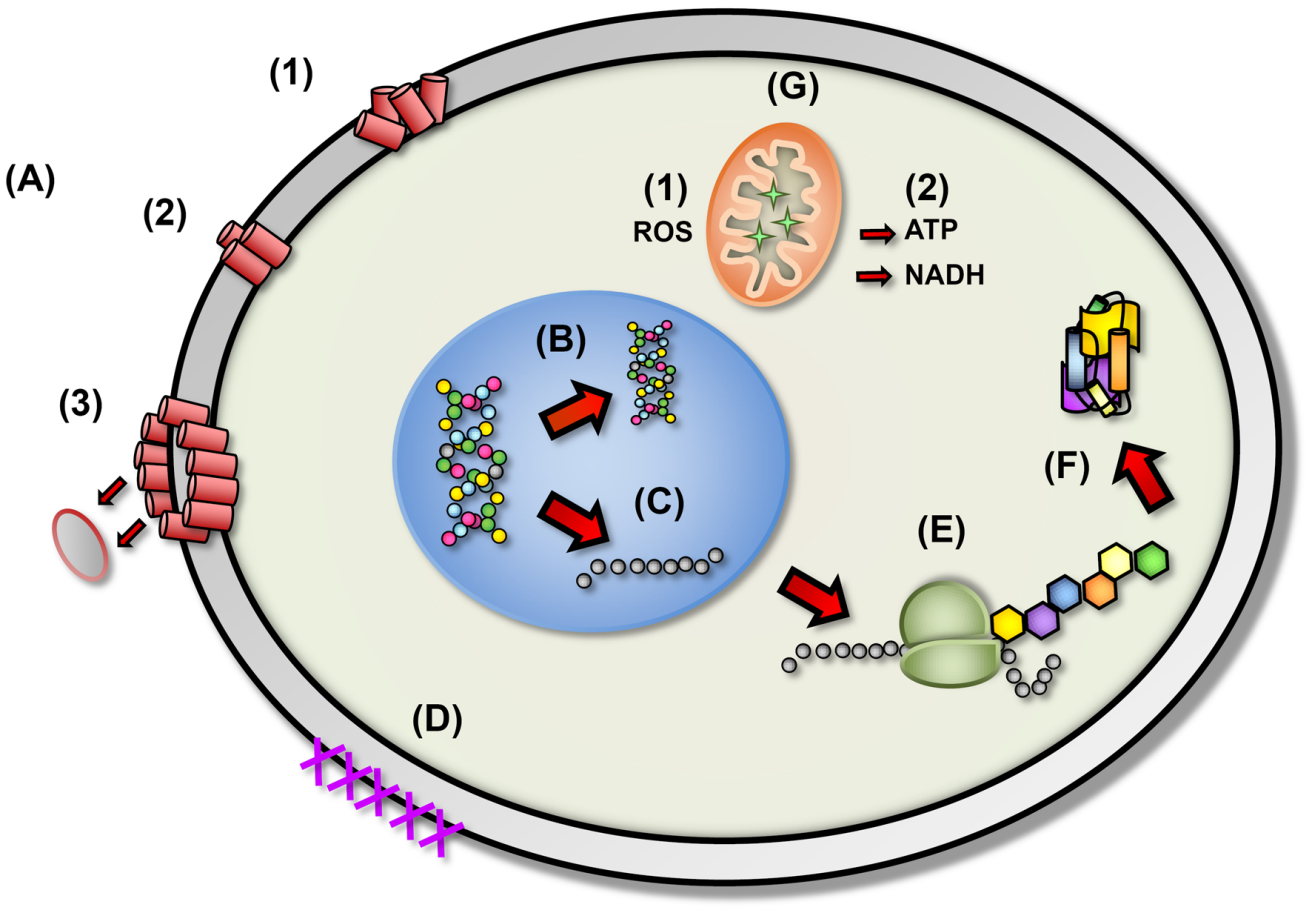
The proposed diverse mechanistic modes of action for antimicrobial peptides in microbial cells. (A) Disruption of cell membrane integrity: (1) random insertion into the membrane, (2) alignment of hydrophobic sequences, and (3) removal of membrane sections and formation of pores. (B) Inhibition of DNA synthesis. (C) Blocking of RNA synthesis. (D) Inhibition of enzymes necessary for linking of cell wall structural proteins. (E) Inhibition of ribosomal function and protein synthesis. (F) Blocking of chaperone proteins necessary for proper folding of proteins. (G) Targeting of mitochondria: (1) inhibition of cellular respiration and induction of ROS formation and (2) disruption of mitochondrial cell membrane integrity and efflux of ATP and NADH.

Despite their vast diversity, most AMPs work directly against microbes through a mechanism involving membrane disruption and pore formation, allowing efflux of essential ions and nutrients. The molecular mechanism and pathway of membrane permeation may vary for different peptides depending on a number of parameters, such as the amino acid sequence, membrane lipid composition, and peptide concentration [3]. Although the mechanisms by which peptides associate with and permeabilize microbial cell membranes are not entirely clear, AMPs are proposed to bind to the cytoplasmic membrane, creating micelle-like aggregates, leading to a disruptive effect (Figure 2). However, a mounting body of evidence indicates the presence of additional or complementary mechanisms such as intracellular targeting of cytoplasmic components crucial to proper cellular physiology (Figure 2) [7], [8]. Thus, the initial interaction between the peptides and the microbial cell membrane would allow them to penetrate into the cell to bind intracellular molecules, resulting in the inhibition of cell wall biosynthesis and DNA, RNA, and protein synthesis. AMPs also possess anti-viral properties, inhibiting viral fusion and egress, thus preventing infection and viral spread via direct interactions with the membranous viral envelope and host cell surface molecules. These properties, combined with the broad range of activity and the short contact time required to induce killing, have led to the consideration of AMPs as excellent candidates for development as novel therapeutic agents. Therefore, insights into the mechanisms employed by AMPs will facilitate new approaches to discover and develop pharmacologic agents.

## Therapeutic Potential

An essential requirement for any antimicrobial agent is that it has selective toxicity for the microbial target, which is an important feature of AMPs, as their preferential interaction with microbial cells makes them non-toxic to mammalian cells. Based on the significant distinctions between mammalian and microbial cells, several factors determine the selectivity of AMPs, such as membrane composition, transmembrane potential, polarization, and structural features [7]–[9]. AMPs have a number of potential advantages as future therapeutics; in addition to their broad spectrum antimicrobial activity and rapid killing of microbes, they neutralize endotoxin and are unaffected by classical antibiotic resistance mechanisms [7], [9], [10]. Significantly, given their proclivity to permeabilize target microbial membranes, the most promising potential application for AMPs is for enhancement of the potency of existing antimicrobials by facilitating access into the microbial cell, resulting in synergistic therapeutic effects [8], [11]. Moreover, unlike conventional antibiotics, which microbes readily circumvent, AMPs do not appear to induce antibiotic resistance, most likely due to the profound changes in membrane structure warranted to confer the microbial cell with resistance. Interestingly, although the fundamental biological role of AMPs is antimicrobial activity, recent studies have highlighted novel alternative functions for these molecules, including immunomodulatory activities, neutralization of endotoxins, wound healing, and anti-neoplastic properties [12]–[16]. Based on the broad and diverse biological functions of these endogenous peptides, AMPs currently are being widely used as blueprints for the development of innovative therapeutic agents that may be used as antimicrobials, modifiers of inflammation, or in cancer therapy.

## Hurdles: Resistance and Immune Evasion

There are several barriers that might impede the development of AMPs as commercial therapeutic agents or restrict their applicability. Among the potential impediments are the high production costs and susceptibility to proteolytic degradation, although AMPs are amenable to extensive chemical modification, which may alleviate some of these obstacles [9]. However, with microbial drug resistance becoming a global public health problem, it has become imperative that new antimicrobials combat the increasing rise in resistance. The development of microbial resistance against AMPs is rare [10]. Nevertheless, microbial pathogens have the capabilities to coordinate countermeasures to circumvent antimicrobial peptide targeting and evade host immune defenses [8], [17], [18].

The newly identified mechanism of immune evasion employed by the pathogenic fungal species *Candida albicans*, the etiologic agent of oral candidiasis (thrush), serves as a good example of microbial strategies to thwart the deleterious effects of AMPs [5]. The salivary antimicrobial peptide histatin-5 exhibits potent anti-candidal properties and therefore is considered to be important in protecting the oral cavity against candidiasis. However, recently *C. albicans* was described to be capable of cleaving histatin-5 not only at specific amino acid residues required for successful intracellular uptake of histatin-5, but also at sites located within the antimicrobial fragment of histatin-5, resulting in deactivation of its anti-candidal potency [19]. This proteolytic ability was attributed to a family of proteolytic enzymes produced by *C. albicans*, the secreted aspartyl proteases, responsible for host tissue degradation and invasion by the fungus [20]. The outcome of this host–pathogen interaction in terms of clinical manifestation was demonstrated to be subject to the ratio of histatin-5 in the saliva to *C. albicans* cell density in the oral cavity [19]. Although *C. albicans* is innately sensitive to histatin-5, compensatory up-regulation of proteolytic enzymes would constitute a potential mechanism for the pathogen to evade host innate defenses [19], [20]. Similarly, the yeast *Candida glabrata* appears to be unaffected by concentrations of AMPs due to increased baseline expression of drug efflux pumps [21].

While acquired resistance to AMPs is less likely to occur as compared to the traditional antimicrobial therapies, some gram-negative bacteria have evolved to utilize various cellular enzymes to biochemically modify and reduce the net negative charge of their cell membranes. By making the net charge more positive, static repulsive forces antagonize insertion of positively charged AMPs into the bacterial membrane [22]. Several other strategies have been described that can result in decreased susceptibility of bacteria to AMPs, such as secretion of inactivating proteins or exportation via efflux pumps. Therefore, we cannot exclude the fact that resistance may evolve if microbial populations are consistently exposed to AMPs. Nevertheless, future research aimed at broadening our understanding of the mechanisms used by both host and microbe will undoubtedly lead to new therapeutic options for managing resistant microbial infections.

## Conclusion

Presently, AMPs represent one of the most promising future strategies for combating infections and microbial drug resistance. This is evident by the increasing number of studies to which these peptides are subjected. As our need for new antimicrobials becomes more pressing, the question remains: can we develop novel drugs based on the design principles of primitive molecules?
